# Sepiolite–Chitosan–Acetic Acid Biocomposite Attenuates the Development of Obesity and Nonalcoholic Fatty Liver Disease in Mice Fed a High-Fat Diet

**DOI:** 10.3390/nu16223958

**Published:** 2024-11-20

**Authors:** Dalia Niv, Eli Anavi, Laris Yaval, Atallah Abbas, Giora Rytwo, Roee Gutman

**Affiliations:** 1 Laboratory of Integrative Physiology, The Department of Nutrition and Natural Products, MIGAL—Galilee Research Institute, P.O. Box 831, Kiryat Shmona 11016, Israel; 2 Environmental Physical Chemistry Laboratory, MIGAL—Galilee Research Institute, P.O. Box 831, Kiryat Shmona 11016, Israel; giorarytwo@gmail.com; 3 Departments of Environmental and Water Sciences, Faculty of Sciences and Technology, Tel-Hai College, Upper Galilee 12210, Israel; 4 Department of Animal Sciences, Faculty of Sciences and Technology, Tel-Hai College, Upper Galilee 12210, Israel

**Keywords:** biocomposite, sepiolite, chitosan, acetic acid, metabolic syndrome, dietary supplement

## Abstract

Background; obesity and nonalcoholic fatty liver disease (NAFLD) reduce life expectancy; nonoperative interventions show poor results. Individually, chitosan (1% *w*/*w*), acetic acid (AA 0.3–6.5% *w*/*w*), and sepiolite clay (5% *w*/*w*) attenuate high-fat-diet-induced obesity (DIO) via reduced energy digestibility and increased energy expenditure. Objectives; therefore, we hypothesized that a chitosan–sepiolite biocomposite suspended in AA would attenuate DIO and NAFLD to a greater extent than AA alone via its more substantial adsorption of nonpolar molecules. Methods; we tested this dietary supplement in C57BL/6J mice fed a high-fat diet (HFD) compared to an unsupplemented HFD and an HFD supplemented with a bile acid sequestrant (cholestyramine) or standalone AA. Results; biocomposite supplementation reduced DIO gain by 60% and abolished hepatic liver accumulation, whereas standalone AA showed mild attenuation of DIO gain and did not prevent HFD-induced hepatic fat accumulation. The biocomposite intake was accompanied by a lower digestibility (−4 point %) counterbalanced by increased intake; hence, it did not affect energy absorption. Therefore, DIO attenuation was suggested to be related to higher energy expenditure, a phenomenon not found with AA alone, as supported by calculated energy expenditure using the energy balance method. Conclusions; these results support further investigation of the biocomposite’s efficacy in attenuating obesity and NAFLD, specifically when applied with a restricted diet. Future studies are needed to determine this biocomposite’s safety, mechanism of action, and efficacy compared to its components given separately or combined with other ingredients.

## 1. Introduction

The prevalence of metabolic syndrome (MetS), which includes diabetes, obesity, atherogenic hypercholesterolemia, hypertension, and nonalcoholic fatty liver disease (NAFLD), is considered to be at pandemic levels [1]. Due to the syndrome’s complexity, drug therapy includes different treatments tackling different mechanisms. For example, obesity is treated by reducing energy (lipid) absorption (e.g., orlistat [2]) or by lowering appetite and increasing energy expenditure (e.g., liraglutide [3]). Similarly, hypercholesterolemia is treated by different mechanisms. For example, statins, which are not well-tolerated at high doses [4], reduce cholesterol production by primarily targeting 3-hydroxy-3-methylglutaryl-CoA reductase in the mevalonate pathway [5]; proprotein convertase subtilisin-kexin type 9 (PCSK9) inhibitors increase the liver uptake of blood LDL-cholesterol by preventing intracellular degradation of LDL receptors [6,7]; and positively charged bile acid sequestrants (BAS), such as cholestyramine, reduce intestinal reuptake of cholesterol-based negatively charged bile salts [6]. For NAFLD, there is no specific approved pharmacological therapy; hence, some of the aforementioned glucose- and lipid-lowering agents have been investigated for NAFLD treatment [7]. Combined therapies suffer from a high potential for drug–drug interactions [8]. Thus, a single drug therapy for MetS would be of high value. Notably, BAS has been recently suggested as a possible treatment for several MetS traits, including hypercholesterolemia, diabetes, obesity, and NAFLD [9,10].

Like BAS, chitosan, a cationic biopolymer produced from chitin and further ionized by acetic acid (AA), has been reported as an indigestible compound that can adsorb bile acids in vitro [11]. In addition, in vivo dietary supplementation of chitosan, even without AA ionization, attenuated high-fat diet (HFD)-induced obesity (DIO), hypercholesterolemia, and hypertriglyceridemia, with a parallel increase in fecal fat and bile acids, without affecting food intake [12,13,14]. This anti-obesity effect is suggested to be mediated by reducing liver lipogenesis and intestinal lipid absorption [15]. However, there is some inconsistency in the reported studies, showing reduced serum cholesterol levels with no increment in fecal bile excretion [16,17]. Moreover, clinical trials have demonstrated similar discrepancies [18,19]. These controversial reports might be explained, at least in part, by an insufficient association between chitosan and bile acids in the gut in vivo, resulting in an altered fecal bile acid profile rather than increased bile acid excretion [16]. Thus, increasing chitosan’s affinity may improve its in vivo efficiency.

The efficiency of chitosan can be increased by generating a chitosan–clay biocomposite (Bc) that exhibits biocompatibility, to serve as a nontoxic biocomposite for drug delivery [20] and tissue engineering [21]. Nevertheless, even clay alone, e.g., montmorillonite, reduces intestinal cholesterol absorption, plasma cholesterol, and obesity rates in vivo [22,23,24,25,26], and was clinically found to be safe for short-term consumption by adults [27,28,29] and children [30]. Using sepiolite, a needle-like clay, may enhance these anti-MetS properties. Sepiolite is a more efficient sorbent than montmorillonite for crude and edible oils [31,32,33,34]. Moreover, in vivo, dietary sepiolite attenuates DIO and lowers blood cholesterol and triglyceride levels, compared to untreated HFD-fed mice, due to higher fecal extraction of lipids and sterols—supporting its role as a gastrointestinal absorber of these compounds [31]. Generating a chitosan–sepiolite biocomposite may further increase sepiolite’s anti-MetS properties by reducing intestinal lipid digestion and reabsorption of the polar anionic bile, as indicated by its ability to attenuate emulsification of an oil–bile salt suspension in vitro due to absorption of the bile salts [35].

Notably, chitosan’s affinity is known to be enhanced by dissolution in a 1% AA solution, resulting in positively charged chitosan biopolymers [36,37]. In vivo, AA also attenuates MetS independently [38,39,40,41,42,43,44]. For example, dietary supplementation of AA results in reduced hepatic triglyceride and fasting glucose levels in diabetic mice [38], inhibition of DIO [39,44], and a decrease in body weight, fat mass, and blood levels of triglyceride and cholesterol in HFD-fed mice [41]; this is due to an increase in energy expenditure and fat oxidation [43], usually with no change in energy intake [38,39,44] (but see [41]). In conclusion, while the effectiveness of the ingredients mentioned above (chitosan, sepiolite, and acetic acid) was tested individually or in pairs, their efficacy as a threesome was never established. The purpose of this work, and, thereby, its innovation, is to close this gap. We hypothesized that a chitosan–sepiolite biocomposite suspended in AA used as a food supplement will obtain a more pronounced attenuation of MetS. We tested this hypothesis in the following proof-of-concept study that explicitly investigated the dose–response effect (0.8% vs. 5% *w*/*w*) of a chitosan–sepiolite–AA (0.8:1:5) biocomposite food supplement in attenuating the development of several MetS traits, such as obesity, diabetes, and dyslipidemia in C57BL/6J mice fed an HFD for 18 weeks. This effect was compared with the impacts of BAS and AA, each administered alone and used as positive controls.

## 2. Materials and Methods

### 2.1. Biocomposite Preparation

A biocomposite suspension of sepiolite (<200 mesh, 99% purity, Tolsa S.A., Madrid, Spain), chitosan (medium molecular weight, 75–85% deacetylated, Sigma-Aldrich Israel, Jerusalem, Israel), and AA (Sigma-Aldrich Israel) was prepared as described in our previous work [45,46] at a 0.8:1 chitosan-to-sepiolite ratio in 1 N AA, giving final concentrations of 2.5% sepiolite and 2% chitosan (*w*/*v*) in 1 N AA. The final solution contained about 10.22% solids (*w*/*v*). The solution was mixed overnight at varying speeds to achieve homogeneity. The chitosan-to-sepiolite ratio of 0.8:1 was chosen because it de-emulsifies gut-like emulsions [24] to a greater extent than other ratios (e.g., 0.06:1, 0.2:1, 0.4:1, 0.6:1, and 1:1) [35,46].

### 2.2. Animals and Diets

The experiment was conducted in full compliance with the strict guidelines of the Israeli Animal Care and Use Committee (permission nos. IL-17-4-161, 5 April 2017, and IL-18-6-173, 13 June 2018). Fifty-four 12-week-old C57BL/6J (B6) male mice (Envigo, Jerusalem, Israel)—a strain that we and others use in similar metabolic studies [31]—were housed individually in a temperature-controlled facility (21–22 °C) with a 12 h light and 12 h dark cycle, and received water and rodent chow (2018S, Envigo Laboratories; energy density of 3.1 metabolizable kcal/g) ad libitum. Following 2 weeks of semi-random acclimation, mice were divided into six groups (n = 9 per group) with similar mean and standard error of the mean (SEM) body weight and body mass composition (i.e., fat percentage, fat mass, and lean mass). This sample size per mouse group was selected according to data obtained in previous experiments [31]. Each group was allocated to be fed ad libitum for 18 weeks on one of six custom-prepared diets (Table 1): (1) a low-fat control diet (LFD, with 9.3% metabolizable kcal from fat; diet composition was based on TD.08806, Envigo); (2) a high-fat control diet (HFD, with 57.8% metabolizable kcal from fat; diet composition was based on TD.06414, Envigo) to induce DIO; (3) an HFD to which 2% (*w*/*w* of dry dietary ingredients) cholestyramine was added (HFD + BAS), to be used as a positive control, having shown attenuated hypercholesterolemia and DIO in B6 mice due to bile acid sequestering [47,48,49]; (4) and (5) two experimental HFDs to which the sepiolite–chitosan–AA biocomposite was added at different doses [a low concentration of 0.8% biocomposite and a high concentration of 5% biocomposite (*w*/*w* of dry dietary ingredients, HFD + BcL and HFD + BcH, respectively)]; and (6) an HFD to which 3% (*w*/*w* of dry dietary ingredients) AA was added (HFD + AA), serving as a control diet to detect the effect of the AA found in BcH.

The high biocomposite dose (5% *w*/*w*; BcH) was selected as it contains 1.2% sepiolite clay, lower than the maximum concentration of 2% in animal feed [50] and similar to the 1.4% dose of montmorillonite clay that has been shown to reduce plasma cholesterol levels in an Apo-E-deficient mouse model with severe hypercholesterolemia (19). BcH also contained 0.8% chitosan, similar to the 1% chitosan found to improve serum lipid profile in rats that consumed a regular diet or a high-fat, high-cholesterol diet [14], and 3% AA—a dose that is midway between the 0.3% and 6.5% doses found to be effective in vivo in reducing MetS symptoms [38,39,40,44]. The low biocomposite concentration (0.8% *w*/*w*; BcL) was chosen in light of preliminary experiments exploring the ratio of active composite to dietary fat that most effectively demulsifies gut-like emulsions [51].

### 2.3. Measurement of Body Weight and Composition, Energy Intake, Absorption, and Digestibility

Body weight, total 24 h food intake, and total 24 h fecal output were recorded weekly. Weekly food intake was used to calculate the average daily food intake. These intakes were used to calculate the respective combustible energy intakes per diet using their combustible energy content measured by bomb calorimetry [52] (Table 2). Feces were stored at −20 °C and oven-dried at 65 °C until they reached a constant weight to determine the total 24 h and cumulative fecal output. Combustible energy content, measured by bomb calorimetry [31] of pooled feces collected in weeks 8, 10, and 14 on a diet (Figure A2A), was used to calculate the apparent absorbed energy and apparent energy digestibility throughout the experiment. These three experimental time points (i.e., weeks 8, 10, and 14 on a diet) were chosen because they represent the period during which most animal groups demonstrated stable body weight and composition (the figure in Section 3.1)—that is, they had established an energy balance. Body composition [fat mass (FM), fat-free mass (FFM, i.e., lean mass), and extracellular fluid (fluid)] was measured every other week using time-domain nuclear magnetic resonance (NMR) (Minispec Analyst AD; Bruker Optics, Silberstreifen, Germany) [53]. Energy intake and absorbed energy were adjusted for body weight and composition differences (i.e., FM and FFM), as performed previously by us and recommended by others [54,55], rather than for body weight, body weight to the power of 0.67 or 0.75, or only FFM. To do so, we defined the mouse’s ‘metabolic mass’ as its FFM plus 18% of its FM, as we found in previous work that the total energy expenditure (TEE) over 24 h of B6 mice fed ad libitum with LFD or HFD equals 0.34 × FFM + 0.06 × FM + 5.16 (R^2^ = 0.66, *p* < 0.01 [55]), i.e., that the covariate of FM is ca. 18% that of FFM.

### 2.4. Estimating Energy Expenditure Using the Energy Balance Method

The energy balance method provides an accurate integrated long-term measurement of TEE in ‘home cages’ while minimizing the potentially confounding stress that may accompany the use of indirect calorimetry systems [56]. According to this method, TEE equals the metabolizable energy intake minus the energy stored or gained from changes (∆) in body composition, calculated as the final minus initial FM and FFM in a given period (Equation (1)).

TEE_bal_ = MEI − (∆FM energy + ∆FFM energy) (1)

We assigned 13.2 kcal for each gram of FM gained, 9.0 kcal for each gram of FM lost, 2.2 kcal for each gram of FFM gained, and 1.0 kcal for each gram of FFM lost [56,57,58]. These changes in somatic energy content were subtracted from the average daily absorbed energy of the given period and adjusted for the average metabolic mass.

### 2.5. Blood Glucose and Serum Lipid Profile

Blood samples were collected after 6 h of fasting from the facial vein (using a lancet) at baseline before replacing the chow diet with the different experimental diets, and again at the experimental endpoint. Blood glucose levels were measured immediately using a hand-held FreeStyle glucose meter (Abbott, Chicago, IL, USA). A ca. 300-µL blood sample was kept on ice until all samples were collected (~3 h); samples were then transferred to room temperature and left to clot for 2 h. The serum was aspirated following centrifugation at 4 °C for 20 min at 1000 RCF and stored at −80 °C. At the experimental endpoint, mice were anesthetized (using isoflurane) for ca. 400-µL blood sampling and sacrificed by cervical dislocation. Blood glucose levels were determined, and serum was collected at baseline. Serum total cholesterol, high-density cholesterol (HDL-C), and triglyceride levels were measured at Rambam Medical Center (Haifa, Israel) and used to estimate low-density cholesterol (LDL-C) and very-low-density cholesterol (VLDL-C) levels.

### 2.6. Measurement of Dietary, Fecal, and Hepatic Lipids and Fecal Bile Acids

Dietary, fecal, and hepatic lipids were extracted using a modified version of a previously published protocol [59]. Briefly, liver biopsies were weighed immediately after dissection (ca. 300 mg), snap-frozen using liquid nitrogen, and stored at −80 °C. Samples were ground in liquid nitrogen and extracted for 2 h at 40 °C using a 4 mL hexane–isopropanol (3:2 *v*/*v*) mixture. The hexane phase was transferred to a 50-mL tube, the extraction procedure was repeated twice, and 2 g sodium sulfate (Chem-Lab NV, Zedelgem, Belgium) was added to absorb water. Following filtration, fluids were transferred to pre-weighed tubes. Dried ground diets and fecal samples (ca. 400 mg each) were processed similarly. For all samples, the total lipophilic content collected from the repeated extractions was measured gravimetrically following the solvent’s evaporation under nitrogen. Fecal lipid content was measured in feces collected at weeks 9, 15, and 16 on a diet (Figure A2A) to calculate the apparent absorbed lipids and their digestibility at these time points and throughout the experiment. These three experimental time points were chosen because they represent the experimental period during which most animal groups demonstrated stable body weight and composition (the figure in Section 3.1)—that is, they established an energy balance.

Fecal bile acids were extracted using a modified version of previously published protocols [60,61]. In brief, ca. 0.5 g oven-dried fecal sample was ground, supplemented with 20 µL of 2000 ppm 5β-cholanic acid (Sigma-Aldrich Israel) as the internal standard, and saponified overnight using 3 mL 0.7 M KOH (Bio-Lab, Jerusalem, Israel). Lipids were first disposed of by two liquid–liquid extraction cycles using hexane (Daejung Chemicals, Siheung-si, Republic of Korea), followed by acidification using 5N HCl (Bio-Lab), and the bile acids were extracted by liquid–liquid extraction using 10 mL chloroform (Bio-Lab). At each extraction phase, which was repeated three times in total, the solution was centrifuged at 4000 RCF for 10 min to separate the phases, and the relevant phase was collected. Water was absorbed using sodium sulfate (Chem-Lab NV), the solution was filtered through #1 Whatman filter paper, and the chloroform was evaporated using nitrogen. Samples were dissolved in 1 mL of dioxane (Sigma-Aldrich Israel).

Bile acid standards cholic acid (CA), lithocholic acid (LCA), deoxycholic acid (DCA), and chenodioxycholic acid (CDCA) (Sigma-Aldrich Israel) were dissolved in dioxane and used to prepare the standard curve by serial dilution in dioxane. Fecal samples and calibration-curve samples (100 µL) were transferred to 2 mL vials, with an insert for gas chromatography–mass spectrometry (GCMS) and 100 µL N, O-bis(trimethylsilyl) acetamide (Sigma-Aldrich Israel), and heated to 80 °C for 1 h. An Agilent Technologies model 7890A System gas chromatograph equipped with an injector with a split device for capillary columns, a chromatographic column (length, 25 m; film thickness, 0.12 µm; inner diameter,0.25 mm; CP-Sil 5 CB, part # CP7710, Agilent, Santa Clara, CA, USA), and a mass spectrometer (5975C, VL MSD with triple-axis detector, Agilent, Santa Clara, CA, USA) was used for all analyses. The GC operating conditions were as follows: a stable flow of 1 mL/min, a pressure of 10.7 PSI, and injector and detector temperatures of 230 °C and 150 °C, respectively. After injection (of 1 µL), the oven temperature was 150 °C, and was then programmed to increase at a rate of 5 °C/min to a final temperature of 280 °C.

### 2.7. Measurement of Hepatic Gene Expression

Liver biopsies (~100 mg) were obtained from sacrificed mice at the experimental endpoint, immediately placed in RNAlater solution (Biological Industries, HaEmek, Israel), and stored at −20° C. Total RNA was isolated using 1 mL TRI Reagent (Thermo Fisher Scientific, Wilmington, NC, USA). After centrifugation (12,000 RCR for 5 min), RNA was extracted with chloroform and precipitated with 0.5 mL isopropyl alcohol (99%). RNA pellets were washed twice in ethanol and resuspended in RNase-free distilled water. RNA quality and concentration were assessed by Nanodrop spectrophotometry (using 260 nm, 280 nm, and 230 nm, Thermo Fisher Scientific). A 1 µg aliquot of extracted RNA in each sample was used to obtain reverse-transcribed cDNA with the Verso cDNA Synthesis Kit (AB1453B, Thermo Fisher Scientific) according to the manufacturer’s instructions, on the Applied Biosystems ABI-2720 Thermal Cycler platform (Thermo Fisher Scientific). Target quantification was performed on the ABI-7000 Sequence Detection System platform using 2x qPCRBIO Fast qPCR SyGreen Blue Mix (PCR Biosystems Inc., Wayne, PA, USA). All primers (see Table 3) were tested for efficiency by serial dilutions, and specificity by melting curve analysis. Each reaction was performed with the thermocycling program according to the manufacturer’s instructions, with a final volume of 20 μL, including 1 μL of cDNA and 200 nM of each primer, in four technical replicates. Results were analyzed using the comparative Ct approach in SDS 2.3 (Thermo Fisher, Warrington, UK), with *GAPDH* as the housekeeping gene, and by Microsoft Excel software.

### 2.8. Hepatic Histopathological Analysis

After dissection, liver biopsies were placed in 4% paraformaldehyde–phosphate buffered saline solution and transferred to overnight incubation at 4 °C with gentle shaking [62]. Samples were rinsed three times in double-distilled water and embedded with paraffin using a dehydration protocol of increasing ethanol concentrations up to 100% and ending in xylene. Tissues were embedded in paraffin and sliced into 6 µm thick sections using a microtome (2030-RM, Leica, Wetzlar, Germany) [62]. Sections were dewaxed, stained with hematoxylin and eosin solution using a standard protocol, and imaged with a Dino-Lite Microscope camera (AnMo Electronics, Taipei, Taiwan) at ×20 magnification [62].

### 2.9. Statistical Analysis

All data are expressed as mean ± SEM. Statistical analysis was conducted using GraphPad Prism v8.0 (La Jolla, CA, USA). The effect of time at experiment and diet on blood glucose and seral lipid and body weight and composition was assessed by two-way repeated measures ANOVA, followed by false discovery rate (FDR) correction for multiple comparisons. The effect of diet on net changes in body weight and composition was assessed by one-way ANOVA followed by FDR correction. One-way ANOVA followed by FDR correction was also used for assessing the effect of diet on parameters related to energy balance (food intake and energy intake and expenditure), energy and lipid digestibility (e.g., their fecal content), fecal bile acids, hepatic lipid accumulation, and hepatic related gene expression related to NAFLD. *p* < 0.05 was considered significant.

## 3. Results

### 3.1. Biocomposite Supplementation Decreases HFD-Induced Fat Gain and Prevents Lean-Mass Loss

Compared to the LFD, the HFD resulted in higher glucose and total cholesterol but lower triglyceride levels (Table 4). The increment in total cholesterol was due to increased HDL-C and LDL-C levels. BAS supplementation prevented HFD-induced hyperglycemia and hypercholesterolemia and further reduced triglyceride levels (Table 4).

**Figure 1:**
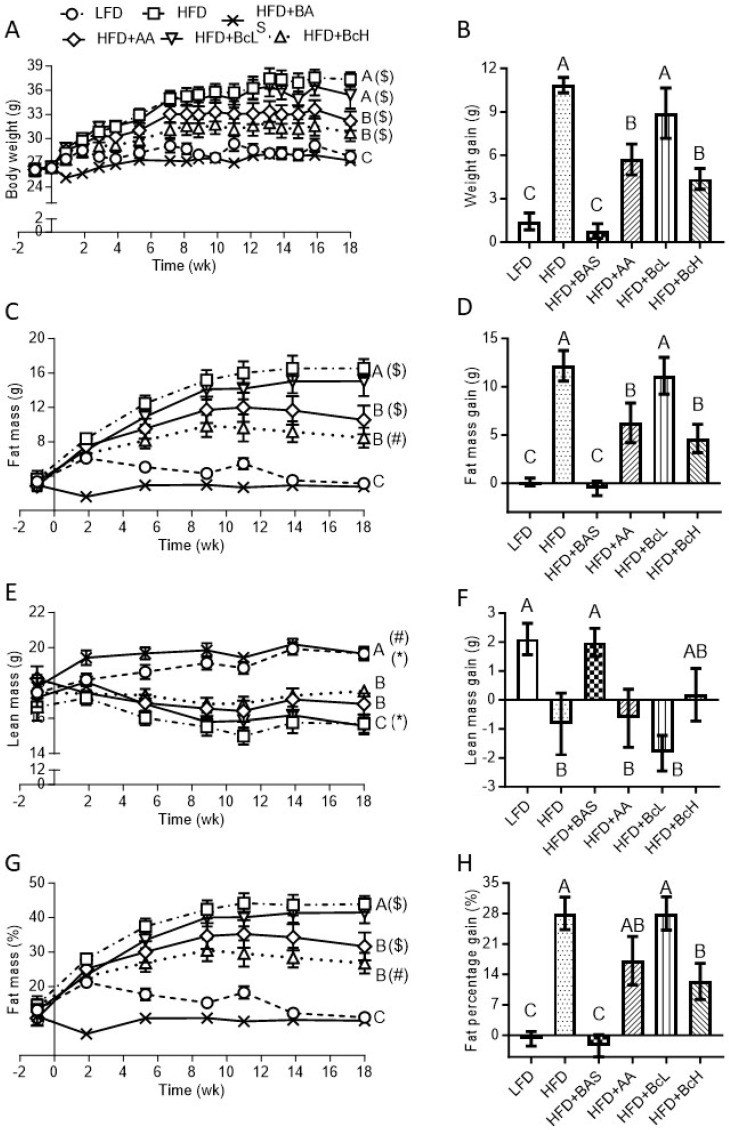
Effect of biocomposite supplementation, compared to control diets, on body weight (**A**), fat mass (**C**), lean mass (**E**), and fat percentage (**G**). Figures on the right (**B**,**D**,**F**,**H**) show the net change in the parameters through the 18-week follow-up. Different letters represent significant differences by FDR post hoc analysis following one-way ANOVA. n = 9 per group. Symbols in parentheses indicate endpoint vs. baseline comparison by FDR post hoc analysis following two-way repeated measures ANOVA, *, *p* < 0.05, ^#^, *p* < 0.01, ^$^, *p* < 0.001. LFD, low-fat diet; HFD + BAS, high-fat diet + 2% (*w*/*w*).

Compared to the LFD, the HFD also caused greater weight gain due to higher fat-mass gain that overcompensated for the reduction in lean mass—the latter was absent with LFD, resulting in a larger fat percentage gain (HFD, 28.04 ± 3.7% vs. LFD, −0.8 ± 1.7, *p* < 0.001, Figure 1H and Table A1). HFD + BAS-fed mice had a lower gain in body weight and fat percentage than mice fed an unsupplemented HFD (BAS, −2.5 ± 2.6% vs. HFD, 28.04 ± 3.7% vs. LFD, −0.8 ± 1.7, *p* < 0.001, Figure 1H and Table A1), resulting in body mass and composition resembling those with LFD feeding (Figure 1). Supplementing BcH to the HFD resulted in a decrease in weight gain (BcH, 4.4 ± 0.7 g vs. HFD, 10.9 ± 0.5 g, *p* < 0.001), but to a lesser extent than the HFD + BAS (*p* < 0.05, Figure 1B and Table A1). The BcH vs. HFD weight-gain difference was explained mainly by the lower fat-mass gain with BcH which, together with the lack of lean-mass loss in HFD + BcH-fed mice, resulted in a lower increment in fat percentage (BcH, +12.4 ± 4.2% vs. HFD, +28.4 ± 3.7%, *p* < 0.05, Figure 1H and Table A1). In other words, this BcH-induced 12.4% gain in obesity (expressed as fat gain percentage) was only 43% of the gain in obesity of the control group (gain of 28.4%).

**Figure A1:**
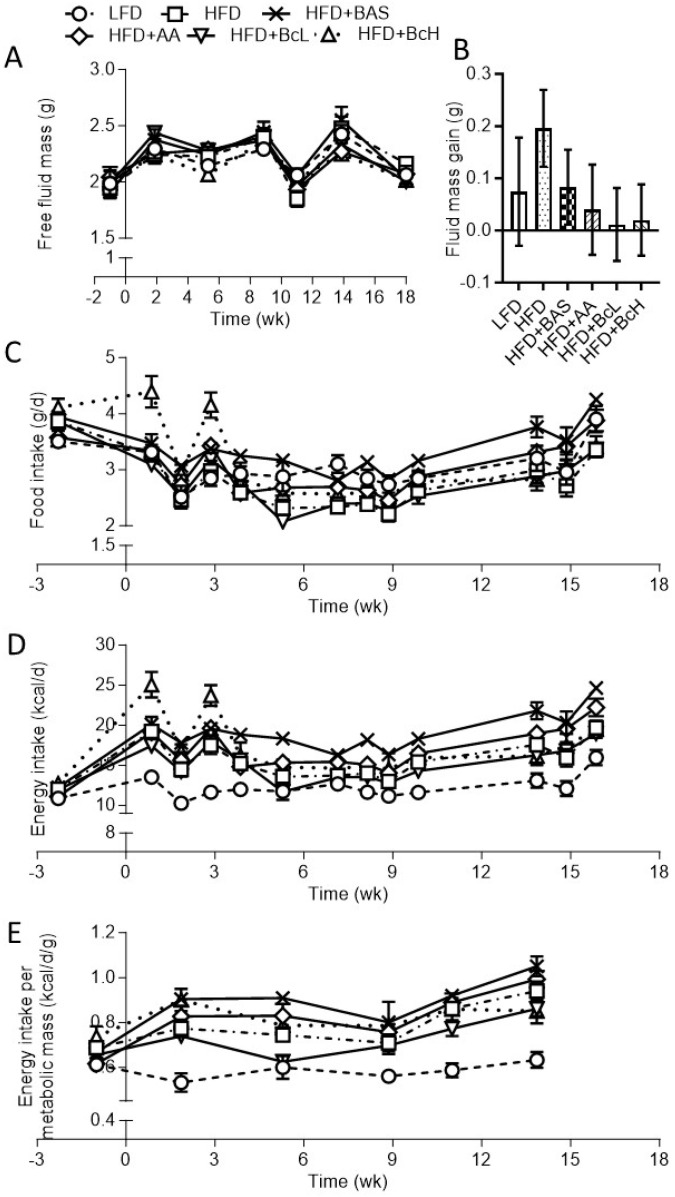
Effect of biocomposite supplementation, compared to control diets, on free fluids (**A**,**B**), food and energy intake (**C**,**D**), and energy intake corrected for metabolic mass (**E**). LFD, low-fat diet; HFD, high-fat diet; HFD + BAS, high-fat diet + 2% (*w*/*w*) cholestyramine; HFD + AA, high-fat diet + 3.0% (*w*/*w*) acetic acid; HFD + BcL, high-fat diet + 0.8% (*w*/*w*) biocomposite; HFD + BcH, high-fat diet + 5.0% (*w*/*w*) biocomposite. n = 9 per group.

Supplementing the HFD with AA (the solvent of the biocomposite) at the dose given to HFD + BcH-fed mice resulted in lower body weight and fat-mass gain than the unsupplemented HFD, as found for BcH (Figure 1 and Table A1). AA supplementation, however, did not prevent the HFD-like lean-mass loss, which differed from the LFD-related lean-mass gain (HFD + AA, −0.6 ± 2.7 g. vs. LFD, 2.1 ± 0.5 g, *p* = 0.05, Figure 1F), contrary to the HFD + BcH that showed an intermitted level of lean-mass gain (0.2 ± 0.9 g, Figure 1F). Taken together, the fat percentage gain under the HFD + AA did not differ statistically from that of the HFD-fed mice (HFD + AA, 17.1 ± 5.6% vs. HFD, 28.0 ± 3.7%, Figure 1H and Table A1). Free fluid content was not affected by diet or time on the diet (age) (Figure A1A). Overall, the −10.9% difference in fat percentage gain under AA (compared to HFD, i.e., 17.1% vs. 28.4%) explained 69.5% of the −15.7% fat percentage gain of the HFD + BcH (compared to the HFD, 12.4% vs. 28.4%, Figure 1H and Table A1). Hence, the effect of the sepiolite–chitosan components accounted for the additional 30.5% of the total impact of the BcH (i.e., the total −17% fat percentage gain of the BcH vs. HFD, Figure 1H and Table A1). This extra effect of the BcH was reflected in the lower fat percentage gain (i.e., lower obesity gain) compared to the unsupplemented HFD, a phenomenon not found in the HFD + AA (Figure 1H and Table A1).

### 3.2. Biocomposite-Induced DIO Attenuation Is Not Due to Reduced Energy Intake or Absorption

**Figure 2:**
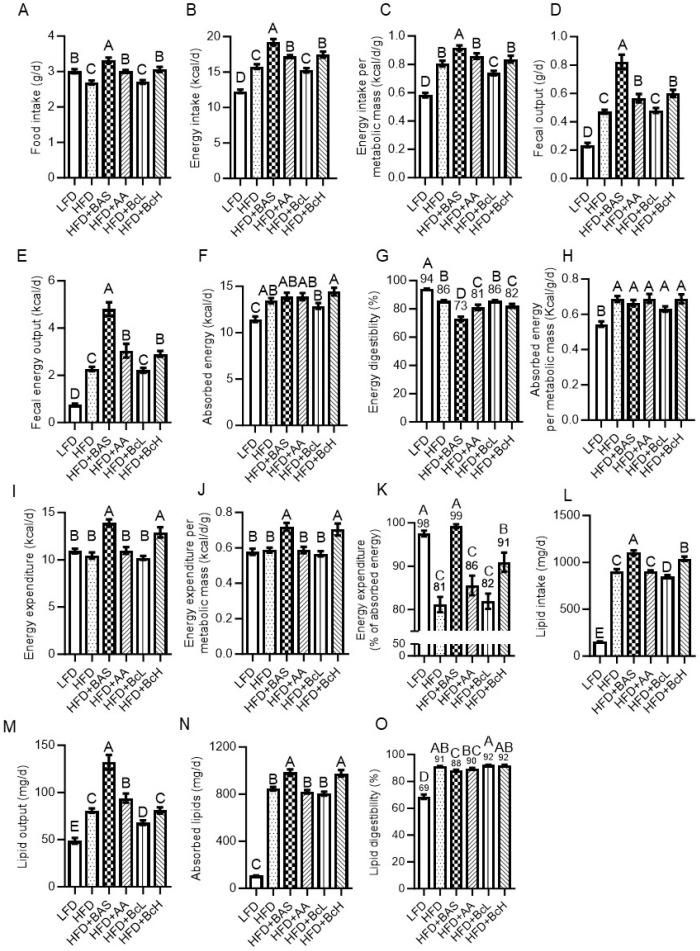
Effect of biocomposite supplementation on energy homeostasis-related parameters compared to control diets. Food and energy intake (**A**,**B**), and energy intake corrected for metabolic mass (**C**); fecal output and fecal energy output (**D**,**E**); absorbed energy and its digestibility (**F**,**G**), and absorbed energy corrected for metabolic mass (**H**); energy expenditure (**I**), corrected for metabolic mass (**J**) and as a percentage of absorbed energy (**K**); and lipid intake (**L**), output (**M**), absorption (**N**), and digestibility (**O**). Different letters represent significant differences by FDR post hoc analysis following one-way ANOVA. n = 9 per group. For abbreviations, see legend in Figure 1.

**Figure A2:**
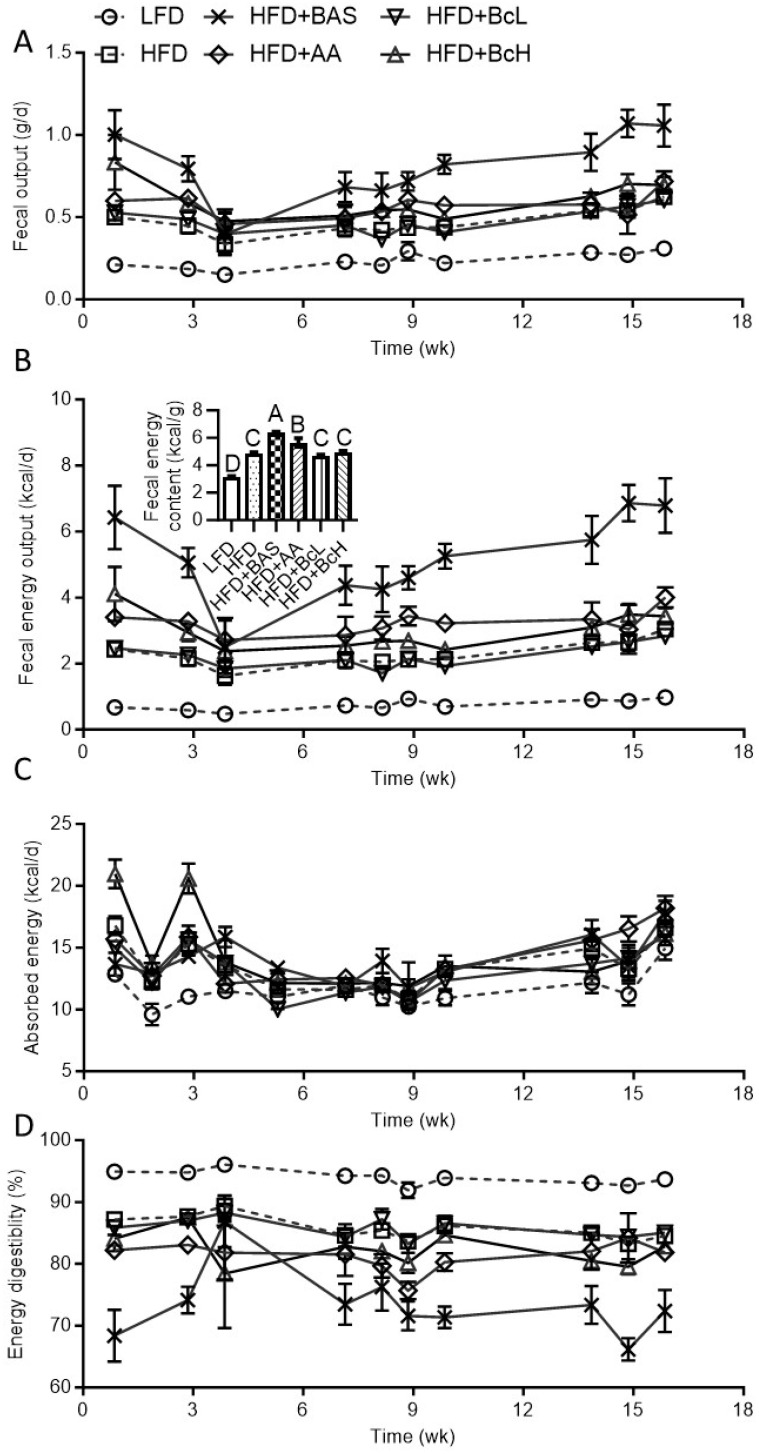
Effect of biocomposite supplementation, compared to control diets, on fecal output (**A**), fecal energy output (**B**), fecal energy content ((**B**), insert), absorbed energy (**C**), and energy digestibility (**D**). Different letters represent significant differences by FDR post hoc analysis following one-way ANOVA. For abbreviations, see legend to Figure A1. n = 9 per group.

Compared to the LFD, the HFD resulted in lower food intake but higher energy intake when corrected for metabolic mass (Figure 2A–C and Figure A1C,D). In parallel, HFD-fed mice also showed higher fecal energy output due to higher fecal production and fecal energy content (Figure 2D,E and Figure A2A,B). However, considering the much higher energy intake, HFD feeding resulted in an overall higher level of absorbed energy (Figure 2F) but lower energy digestibility than the LFD (HFD, 86 ± 0.4% vs. LFD, 94 ± 0.4%, *p* < 0.001, Figure 2G and Figure A2D). This higher absorbed energy in HFD-fed mice was even more pronounced when corrected for metabolic mass (Figure 2H)—energetically supporting their more positive energy, i.e., their DIO, compared to LFD-fed mice (Figure 1B,H). Compared to the unsupplemented HFD, the HFD supplemented with BASs, AA, or the BcH resulted in higher rather than lower food intake and, therefore, higher energy intake, even when corrected for metabolic mass in the case of BAS-supplemented mice (Figure 2A–C and Figure A1C–E). These results suggest that DIO attenuation in BAS-, AA-, and BcH-fed mice is due to attenuated energy absorption and/or higher energy expenditure, rather than attenuated energy intake.

In parallel, these three mouse groups showed higher fecal energy output due to higher fecal production and fecal energy content (except for BcH) than HFD-fed mice (Figure 2D,E and Figure A2). However, considering their energy intake, they showed an HFD-like level of absorbed energy, indicating decreased energy digestibility (BASs, 73 ± 1.5%; AA, 81 ± 1.9%; and BcH, 82 ± 1.4% vs. HFD, 86 ± 0.4%, Figure 2G and Figure A2). This HFD-similar absorbed energy was also found when corrected for metabolic mass (Figure 2H). In parallel, BAS-fed mice showed higher energy intake and absorbed energy (also when corrected for metabolic mass) than LFD-fed mice (Figure 2C,H). These results indicate that BAS supplementation does not reduce energy intake and can even increase it, followed by lower digestibility, resulting in an HFD-like energy absorption rate (Figure 2C,F–H). Hence, the attenuation of DIO by BAS supplementation is proposed to be due to higher metabolic-mass-corrected energy expenditure (see Section 3.3). This would also explain the LFD-like body mass and composition of HFD + BAS-fed mice, which also showed a more elevated (metabolic-mass-corrected) energy intake and absorption rate than LFD-fed mice (Figure 2C,H). Similarly, the attenuated DIO of AA- and BcH-fed mice is suggested to be due to higher energy expenditure rather than reduced energy intake or absorption rates.

### 3.3. DIO Attenuation Is Related to Higher Energy Expenditure, as Calculated by the Energy Balance Method

Using the change in fat and lean mass during the first 9 weeks of the experiment—after which body composition stabilized (Figure 1)—we calculated the total energy lost or gained (during tissue deposition or catabolism) per diem. This lost or gained energy was subtracted from the per diem absorbed energy (Figure 2F) to give the TEE over 24 h (Figure 2I). DIO did not affect this metabolic-mass-corrected TEE (Figure 2J and Table A1). In contrast, as suggested above, the HFD + BAS and HFD + BcH resulted in higher-than-expected energy expenditures than the HFD (Figure 2J and Table A1). More specifically, HFD-fed mice expended 81 ± 1.8% of their absorbed energy (vs. 98 ± 0.7% for LFD-fed mice, Figure 2K). The remaining 19% was used for and stored as body tissue, mainly fat mass (Figure 1). At the same time, the supplementation of the HFD with BASs resulted in LFD-like usage of energy intake (TEE = 99 ± 0.5% of intake, Figure 2K). Supplementation of the HFD with the BcH resulted in an intermediate value of 91 ± 2.3%, higher than that of the HFD + AA (86 ± 2.3%, *p* < 0.05, Figure 2K). These findings support the notion that DIO is due to higher energy absorption rather than reduced energy expenditure. At the same time, the DIO attenuation rate by the different dietary supplements was related to how closely the increased energy expenditure matched the HFD-induced increment in absorbed energy (Figure 2K).

### 3.4. DIO Attenuation by Biocomposite Supplementation Is Not Due to Attenuated Lipid Digestibility

**Figure A3:**
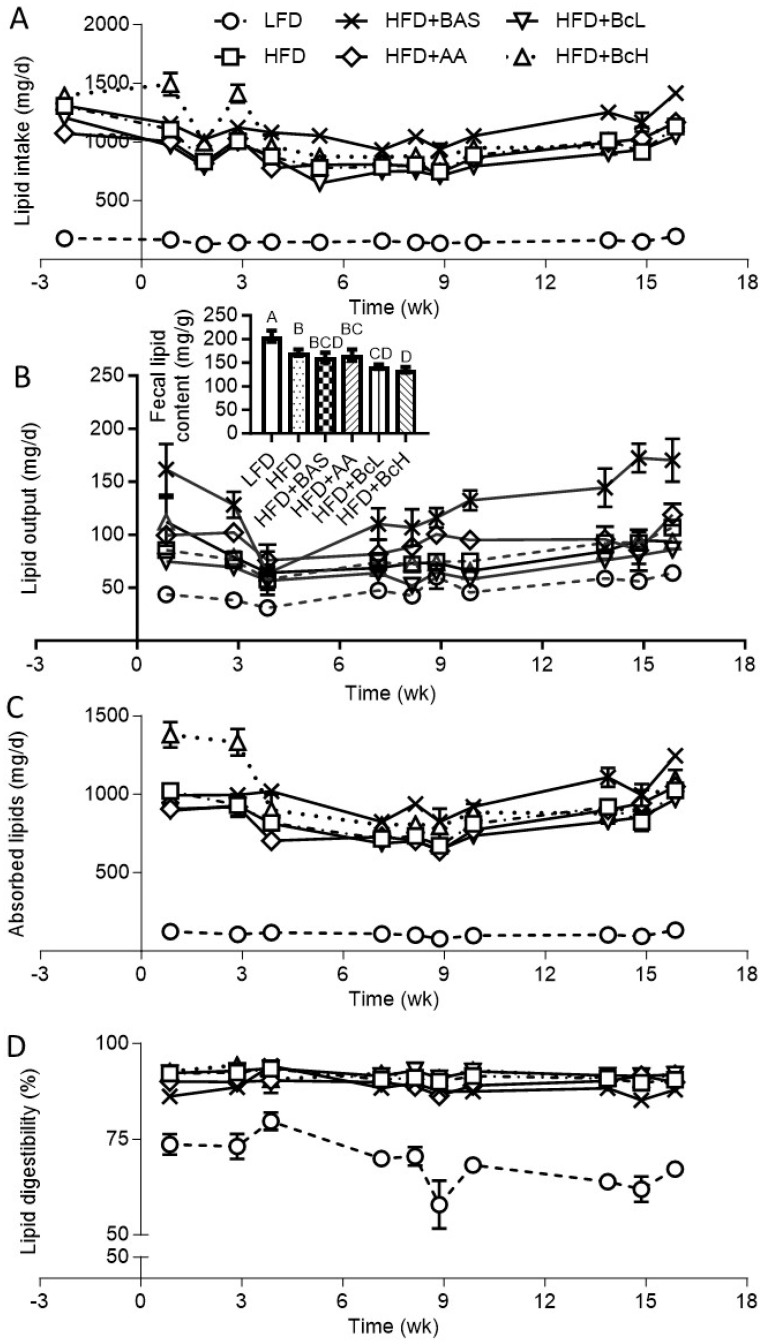
Effect of biocomposite supplementation, compared to control diets, on lipid intake (**A**), fecal lipid output (**B**), fecal lipid content ((**B**), insert), absorbed lipids (**C**), and lipid digestibility (**D**). Different letters represent significant differences by FDR post hoc analysis following one-way ANOVA. n = 9 per group. For abbreviations, see the legend in Figure A1.

Compared to the LFD, feeding on the HFD resulted in higher lipid intake, as found for energy intake, due to the HFD’s higher lipid content, which counterbalanced the HFD-induced lower food intake (Table 1, Figure 2L and Figure A3A). HFD-fed mice also showed higher lipid output due to the higher fecal output that counterbalanced their lower fecal lipid content (Figure 2M and Figure A3B, insert). However, the HFD resulted in a higher final level of absorbed lipids, considering the much higher lipid intake, also resulting in higher lipid digestibility than in LFD-fed mice (HFD, 91 ± 0.3% vs. LFD, 69 ± 1.7%, *p* < 0.01, Figure 2N,O and Figure A3C,D). This HFD-induced increment in lipid digestibility suggests that the above-reported HFD-induced reduction in energy digestibility (Figure 2G) is due to an HFD-induced decrease in non-lipid energy digestibility.

**Figure 3:**
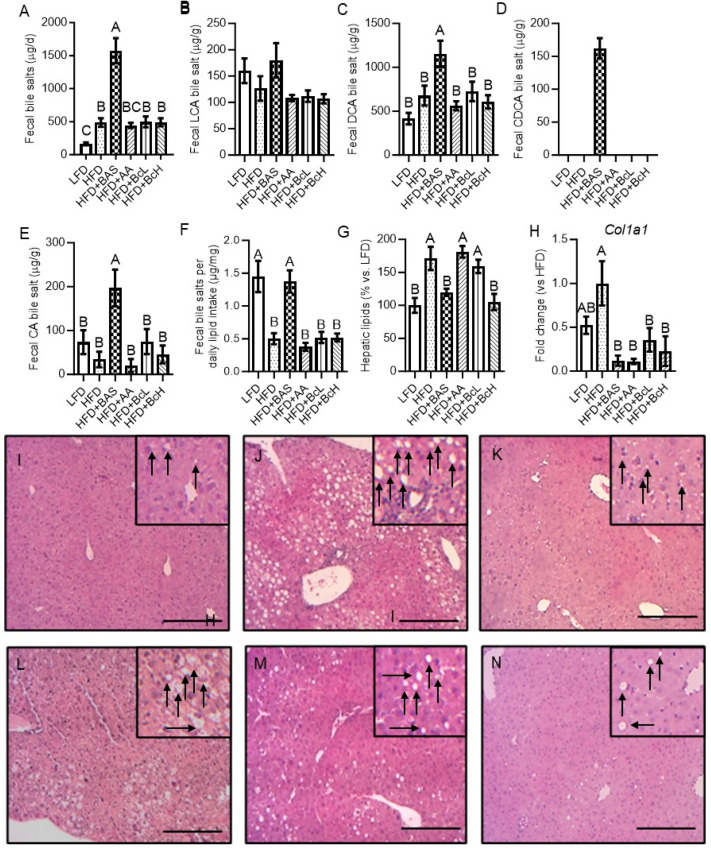
Effect of biocomposite supplementation, compared to control diets, on daily output and composition of fecal bile salts (**A**–**E**), and corrected to lipid intake (**F**); hepatic lipid content (**G**); mRNA expression of collagen type 1 gene (Col1a1, (**H**)); and hepatic histology ((**I**–**N**); (**I**), LFD; (**J**), HFD; (**K**), HFD + BAS; (**L**), HFD + AA; (**M**), HFD + BcL; and (**N**), HFD + BcH). Hepatic sections were imaged at ×20 magnification. Scale bar, 200 µm. Arrows indicate lipid droplets. Different letters represent significant differences by FDR post hoc analysis following one-way ANOVA. n = 8–9 per group for bile data, n = 6 for hepatic histology and gene expression. LCA, lithocholic acid; DCA, deoxycholic acid; CDCA, chenodeoxycholic acid; CA, cholic acid. For other abbreviations, see the legend in Figure 1.

As found for energy intake, the HFD supplemented with BASs, AA, or the BcH resulted in a higher or similar lipid intake compared to the unsupplemented HFD (Figure 3L and Figure A3A). In parallel, HFD supplementation with BASs resulted in a higher output of fecal lipids due to higher fecal output; the fecal lipid content was unaffected (Figure 2M and Figure A3B). Considering lipid intake, however, the HFD + BAS- and HFD + BcH-fed mice showed higher levels of lipid absorption than those fed an HFD (Figure 2N and Figure A3C). Taken together, lipid digestibility was not affected by any of the supplements, except for lower digestibility with BAS supplementation (Figure 2O and Figure A3D). This indicates that the higher lipid absorption under the HFD supplemented with BAS or the BcH (Figure 2N) was due to the higher food (lipid) intake (Figure 2A,L) rather than increased digestibility. These results also indicate that the observed attenuations in DIO are not solely due to attenuation of lipid digestibility or absorption; they also need to counterbalance their accompanied increment in lipid absorption (due to increased food intake). Moreover, as BAS and the BcH show a more subtle reduction (if any) in lipid digestibility than the reduction in total energy digestibility (Figure 2O vs. Figure 2G), their consumption is also suggested to induce a decrease in non-lipid energy digestibility.

### 3.5. Biocomposite Supplementation Does Not Affect the HFD-Induced Increment in Bile Salt Extraction Rate

Compared to the LFD, the HFD resulted in a higher fecal output of bile salts due to the HFD-induced increment in fecal production (Figure 2D and Figure 3A), while the fecal content of bile salts was unaffected by diet (Figure 3B–E). However, this higher loss in fecal bile salts was lower than expected, according to the increment in lipid intake (Figure 3F). The HFD + BAS resulted in an even higher fecal output of bile salts due to the BAS-induced increment in fecal production and fecal DCA, CDCA, and CA concentrations (Figure 2D and Figure 3A–E). This loss in fecal bile salt was higher than expected according to the lipid intake (Figure 3F), indicating its association with BAS per se and not its associated increment in lipid intake (Figure 2L). The BcL, BcH, and AA did not affect fecal bile-salt concentration or output (Figure 3A–E).

### 3.6. Biocomposite Supplementation Attenuates HFD-Induced Hepatic Lipid Accumulation and Related Gene Expression

Compared to the LFD, the HFD resulted in the accumulation of hepatic lipids, detected by lipid extraction and also shown by hematoxylin and eosin staining, suggesting the development of NAFLD (Figure 3G,I,J and Table A1). Compared to the HFD, the HFD supplemented with either BAS or the BcH demonstrated lower lipid content and tissue morphology similar to the LFD (Figure 3G,I–N and Table A1). Hepatic mRNA-expression analysis of Col1a1, which encodes for collagen-type 1—a gene related to the development of hepatic fibrosis [63,64]—showed that compared to the LFD, HFD-feeding resulted in a trend toward higher expression (Figure 3H). This trend was reversed by all food supplements, showing lower Col1a1 levels than the HFD-fed mice (Figure 3H).

## 4. Discussion

Numerous in vivo and a few human trials have shown the effectiveness of standalone AA [38,39,40,41,42,43,44], chitosan [12,13,14,16,17], and edible montmorillonite clay [22,23,24,25,26] as food supplements attenuating HFD-induced MetS; however, conflicting results have been obtained in clinical studies [18,19,65,66,67,68]. In addition, we recently showed this anti-DIO and hypercholesterolemia effect for edible sepiolite clay [31]. In the present study, we examined the anti-MetS effectiveness of a food supplement combining these three substances, i.e., a biocomposite combining sepiolite, chitosan, and AA, the latter also ionizing the chitosan for a positively charged biocomposite, which was hypothesized to serve as a BAS. BcH supplementation attenuated DIO gain and NAFLD development more than was found with standalone AA but less than cholestyramine (a known BAS). BcH resulted in a higher intake that counterbalanced its associated reduced energy digestibility, and a higher energy expenditure—mechanistically explaining the observed amelioration in MetS.

Compared to the LFD, HFD feeding resulted in MetS: obesity, hypercholesterolemia, NAFLD, and hyperglycemia, as also shown in other studies [10,31,53,55,69], but paradoxically, it decreased circulating triglycerides, as also demonstrated by others [70,71,72]. This HFD-induced reduction in seral triglycerides is probably due to increased seral triglyceride clearance and its accumulation in the liver (see HFD-induced NAFLD), as suggested by others [70,71,72]. The hypercholesterolemia was due to increased LDL-C and HDL-C, as found for C57BL6 mice chronically fed an HFD for 10–16 weeks [73,74,75]. The DIO was underlined by increased adiposity and lean-mass loss and was explained energetically by a higher-than-expected (by metabolic mass) energy intake rather than reduced energy expenditure, as we have shown previously [55]. This higher energy intake, which overcompensated for the known (see [76]) HFD-induced increment in fecal energy output and reduced energy digestibility, resulted in a higher (metabolic-mass-corrected) level of absorbed energy. As expected, BAS supplementation to the HFD prevented DIO, NAFLD, hyperglycemia, and hypercholesterolemia [47,77,78,79,80,81]. The parallel increment in fecal bile acid loss, which exceeded the HFD-induced increment in bile acid loss and was higher than expected based on lipid intake, explains the BAS-induced reduction in serum cholesterol, as also suggested by others [79,81]. As previously reported [79], the HFD + BAS also resulted in a higher energy intake, paralleling the reduced energy digestibility, yet it did not affect energy or lipid absorption compared to the HFD; hence, the BAS-induced stabilized energy balance can be explained by higher metabolic-mass-corrected energy expenditure, as shown by others [47,78]. The above-described similarities between our and others’ results validate the methods and approaches used to obtain the subsequent results.

Supplementing the HFD with the BcH attenuated DIO and NAFLD progression more so than AA alone, which has been previously shown to attenuate diet-induced MetS [38,39,40,41,42,43]. The BcH-induced attenuation of the HFD-induced increment in adiposity percentage was due to attenuated fat-mass gain and prevention of lean-mass loss, resulting in a leveled lean mass that did not differ from the LFD-related lean-mass gain. In contrast, standalone AA only showed the expected fat-gain attenuation [39]. The trend of a lower fat percentage gain under AA (compared to HFD alone) accounted for 70% of the total effect observed under the HFD + BcH. The BcH, however, also resulted in significantly lower fat percentage gain than the HFD (explaining the added 30% difference between the HFD + BcH and HFD) due to its more extensive prevention of lean-mass loss and fat-mass gain. The BcH is also advantageous over AA in preventing the HFD-induced increase in hepatic lipids (a symptom of NAFLD), a prevention found only with the HFD + BcH and HFD + BAS but not with the HFD + AA consumption. These dietary attenuations in NAFLD progression were paralleled by lower mRNA expression levels of Col1a1, which encodes for collagen type 1 and is related to developing hepatic fibrosis [63,64], than found for HFD-fed mice.

BcH supplementation did not attenuate energy intake, as found for AA supplementation in this and other studies [38,39]. BcH supplementation was accompanied by reduced energy digestibility (not attributed to a decrease in lipid digestibility) that, however, did not attenuate energy or lipid absorption due to the parallel increment in their intake. These effects were attributed to the AA supplementation and were unaffected by the additional sepiolite and chitosan found in the BcH. As the BcH did not affect absorbed energy, it is suggested that the BcH-induced attenuation in DIO is due to elevated energy expenditure and increased fat oxidation, to an even greater extent than seen for AA alone [43]. Our energy expenditure estimation using the energy balance method supports this suggestion; the BcH and BAS, the only dietary supplements that significantly attenuated fat percentage gain, showed a higher-than-expected energy expenditure than HFD-fed mice. Hence, the additive effect of sepiolite and chitosan found in the BcH seems to be the increment in energy expenditure. This biocomposite-induced elevation in energy expenditure could be underlined by changes in several metabolic pathways, such as increased hepatic expression of the JAK2-STAT3 signaling pathway to alleviate leptin resistance and increased expression of browning genes and proteins (e.g., UCP1, PGC1α, PRMD16, and ATF2) in white and brown adipose tissues [82,83]. Nevertheless, this result needs to be validated by indirect calorimetry, a method applied in our previous studies [55,69,84]; this is planned for future studies.

Contrary to our hypothesis that the BcH can effectively adsorb bile salts in vivo and therefore increase their fecal extraction, the feeding of the HFD + BcH was not accompanied by higher fecal bile salts than the HFD. This between-diet similarity in fecal bile salt content goes hand in hand with their similarity in blood cholesterol levels, in contrast to the (expected, see [47,48,49]) findings for the HFD + BAS showing higher fecal bile salt loss in parallel with lower blood cholesterol levels than the HFD. In addition, our current results do not support previous ones showing that supplementing sepiolite clay alone to a high-fat, high-cholesterol diet results in attenuated hypercholesterolemia in addition to attenuated DIO [31]. That study, however, used 5% (*w*/*w*) sepiolite—twice the sepiolite concentration used in the current study, a difference that could at least partly explain the discrepancy in the results. Moreover, in that previous study, hypercholesterolemia was induced by adding dietary cholesterol to be absorbed by the uncharged lipophilic sepiolite [31]. This study, however, aimed at attenuating hypercholesterolemia by absorption of the cationic fecal bile salts by the presumably anionic biocomposite. Our current results, which lack a BcH-induced elevation in fecal bile salts, suggest that the biocomposite lost its AA-conferred in vitro cationic properties [35] while passing through the gastrointestinal tract. This effect might be induced by the duodenal buffers that maintain a pH of ca. 7 [85]—a pH at which our biocomposite might lose its cationic properties [35]. This issue will be explored in future studies.

An updated meta-analysis showed that even novel anti-obesity drugs prescribed alongside a control diet show a placebo-subtracted weight reduction that does not exceed 7% over 12 months [2]. Moreover, the only long-term effective over-the-counter oral medication for obesity is orlistat, with an efficacy of up to 3% weight reduction [2], but it does not consistently reverse liver fibrosis [86]. Our results show that supplementing an HFD with BcH attenuates body weight gain by 18 ± 2.8%, even under ad libitum feeding, while simultaneously addressing NAFLD, which was not attenuated under AA.

While the safety of chitosan and AA consumption has been clinically addressed [18,19,65,66,67,68], the safety of clay consumption is the subject of an ongoing investigation. In this preliminary safety assessment, dietary sepiolite (1.2% *w*/*w*), part of the BcH, did not appear to cause notable adverse effects, such as chronic fatty diarrhea (steatorrhea). This dose is lower than the maximum 2% concentration of sepiolite (E562) allowed in animal feed by the European Food Safety Authority (EFSA) [50] and four times lower than the 5% dose (*w*/*w*) used in our previous study with no notable adverse effects [31]. Further analyses of, for example, seral biochemical indexes and liver histopathology are needed to approve the safety of sepiolite consumption. In addition, the long-term efficacy and safety of biocomposite supplementation should also be studied in human trials.

## 5. Conclusions

In conclusion, we show that continuous supplementation of a sepiolite–chitosan–AA biocomposite (5% *w*/*w*) to an HFD attenuates its MetS-related outcomes, including fat-mass gain, lean-mass loss, and NAFLD (i.e., hepatic lipids accumulation). Standalone AA, however, showed mild attenuation of DIO gain and did not prevent HFD-induced hepatic fat accumulation. Mechanistically, BcH intake was accompanied by reduced energy digestibility counterbalanced by increased intake; hence, it did not affect energy absorption. Therefore, DIO attenuation was suggested to be related to higher energy expenditure, a phenomenon not found with AA alone, as supported by calculated energy expenditure using the energy balance method. Hence, these results support further investigation of the biocomposite’s efficacy as a food supplement that can further attenuate the development of MetS and may do so to a greater extent if applied with a restricted diet. Future studies should also focus on the safety of this biocomposite, such as in terms of seral biochemical indexes and liver histopathology. The mechanism of action should also be further studied, and, for example, the suggested elevated energy expenditure should be evaluated using indirect calorimetry. Lastly, the biocomposite’s efficacy should be investigated and compared to its three components, given separately or in combination with the others, to evaluate any synergistic effect of the components.

## Figures and Tables

**Table 1:** Formulation of the custom-prepared diets.

Formulation			Diets (g/kg of Ingredients)					
Ingredient	Manufacturer, Catalog No.	Humidity (%) ^1^	LFD	HFD	HFD + BAS ^2^	HFD+ AA ^3^	HFD+ BcL ^4^	HFD + BcH ^4^
Casein	Frutarom (Haifa, Israel), 9500682599	7.5	210	265	265	265	265	265
L-Cysteine	MP Biomedicals (Irvine, CA, USA), 210144490	<0.1	3	4	4	4	4	4
Maltodextrin	Zhucheng Dongxiao Biotec. (Jiaozhou City, Shandong, China)	4.6	465	160	160	160	160	160
Cornstarch	Galam (Maanit, Israel)	7.8	100	0	0	0	0	0
Sucrose	Sugat (Kiryat Gat, Israel), 290000211503	<0.1	90	90	90	90	90	90
Beef fat	Local slaughterhouse	7.3	20	310	310	310	310	310
Soybean oil	Supersal (Rishon LeTsiyon, Israel), 2900024317	<0.1	20	30	30	30	30	30
Cellulose	MP Biomedicals, 0219149991	5.9	37.25	65.6	65.6	65.6	65.6	65.6
Mineral mix	MP Biomedicals, 0296040002	0.3	35	48	48	48	48	48
Calcium phosphate	Sigma (Jerusalem, Israel), c7263	0.1	2	3.4	3.4	3.4	3.4	3.4
Vitamin mix	MP Biomedicals, 0296040201	0.8	15	21	21	21	21	21
Choline bitartrate	MP Biomedicals, 0210138483	0.1	2.75	3	3	3	3	3
Total ingredients (g)			1000	1000	1000	1000	1000	1000

Abbreviations: LFD, low-fat diet based on TD.08806 (Envigo); HFD, high-fat diet based on TD.06414 (Envigo); HFD + BAS, HFD + 2% (*w*/*w*) cholestyramine; HFD + AA, HFD + 3.0% (*w*/*w*) acetic acid; HFD + BcL, high-fat diet + 0.8% (*w*/*w*) biocomposite; HFD + BcH, high-fat diet + 5.0% (*w*/*w*) biocomposite. ^1^ Humidity in ingredients (%) was measured by oven-drying for at least 72 h or until they reached constant weight at 65 °C. ^2^ Bile acid sequestrant (cholestyramine). ^3^ Acetic acid. ^4^ Chitosan and sepiolite at a 0.8:1 ratio in 1 N acetic acid, resulting in final concentrations of 2.5% sepiolite and 2% chitosan (*w*/*v*) in 1 N acetic acid.

**Table 2:** Dietary compounds and nutritional composition of the custom-prepared diets.

Supplemented Compounds, on Top (g)	Humidity (%)	LFD	HFD	HFD + BAS	HFD + AA	HFD + BcL	HFD + BcH
Cholestyramine (2% *w*/*w*)	7			20 ^1^			
Acetic acid, 1 N (3% *w*/*w*)	94.0				480 ^2^		
Biocomposite ^3^ (0.8% *w*/*w*)	98.3					80 ^3^	
Biocomposite ^3^ (5% *w*/*w*)	90.0						480 ^3^
Added water		540	80	80			
Total weight (g)		1540	1080	1100	1480	1080	1480
Selected nutritional information							
Humidity in diet (%) ^4^		20.8	11.5	8.7	14.1	11.2	11.1
Carbohydrates (%, *w*/*w*) ^5^		65.8	28.9	28.4	28.9	28.7	27.7
Protein (%, *w*/*w*) ^5^		20.7	26.3	25.8	25.6	26.1	25.1
Fat (%, *w*/*w*) ^5^		4.1	33.5	32.9	32.6	33.3	31.9
Carbohydrates (% of MEI) ^6,7^		69.5	22.1	22.1	21.8	22.1	21.9
Protein (% of MEI) ^6,7^		21.2	20.1	20.1	19.9	20.1	19.9
Fat (% of MEI) ^6,7^		9.3	57.8	57.8	58.2	57.8	58.2
Metabolizable energy (kcal/g) ^7^		3.9	5.2	5.1	5.1	5.2	5.0
Combustible energy (kcal/g; mean ± SD) ^8^		4.1 ± 0.31	5.9 ± 0.25	5.8 ± 0.07	5.7 ± 0.1	5.6 ± 0.29	5.7 ± 0.15
Lipids (%) ^9^		5.1 ± 0.98	33.8 ± 0.3	33.3 ± 0.3	30.1 ± 0.3	31.3 ± 0.3	34.0 ± 0.5

Abbreviations: LFD, low-fat diet based on TD.08806 (Envigo); HFD, high-fat diet based on TD.06414 (Envigo); HFD + BAS, HFD + 2% (*w*/*w*) cholestyramine; HFD + AA, HFD + 3.0% (*w*/*w*) acetic acid; HFD + BcL, HFD + 0.8% (*w*/*w*) biocomposite; HFD + BcH, HFD + 5.0% (*w*/*w*) biocomposite; MEI, metabolizable energy intake. ^1^ Bile acid sequestrant (cholestyramine). ^2^ Acetic acid. ^3^ Chitosan and sepiolite at a 0.8:1 ratio in 1 N acetic acid, resulting in final concentrations of 2.5% sepiolite and 2% chitosan (*w*/*v*) in 1 N acetic acid. ^4^ Humidity in ingredients (%) was measured by oven-drying for at least 72 h or until they reached constant weight at 65 °C. ^5^ Calculated for 1 kg final dry diet, including food supplements, considering the humidity in each ingredient and the total diet. ^6^ Calculated percentage of metabolizable energy intake (kcal) in the final dry diet considering the humidity in each ingredient and the total diet. ^7^ Calculated metabolizable energy content, considering the humidity in each ingredient, including food supplements, in the final diet. Estimated based on Atwater factors assigning 4 kcal/g to dry protein, 9 kcal/g to dry fat, 4 kcal/g to dry available carbohydrate, 0.89 kcal/g to dry mineral mix as it contains 22.1% sucrose, 3.84 kcal/g to dry vitamin mix as it contains 96.0% sucrose, and 0.11994 kcal/g 1 N acetic acid. ^8^ Combustible energy content, measured by bomb calorimetry following oven-drying for at least 72 h or until it reached constant weight at 65 °C. ^9^ Dietary lipids, measured by extraction for 2 h at 40 °C using a hexane–isopropanol (3:2 *v*/*v*) mixture.

**Table 3:** List of primers used for quantitative real-time PCR.

Gene	Forward Primer	Reverse Primer
*Col1a1*	GCTCCTCTTAGGGGCCACT	CCACGTCTCACCATTGGGG
*Gapdh*	GGTCTACATGTTCCAGTA	CCCATTTGATGTTAGTGG

**Table 4:** Characteristics of mice’s blood glucose and seral lipid levels (mg/dL).

Parameter	Time Point	LFD	HFD	HFD + BAS ^1^	HFD + AA ^2^	HFD + BcL ^3^	HFD + BcH ^3^
Glucose	Baseline	130.3 ± 5.0	130 ± 5.5	129.8 ± 7.9	137.8 ± 6.7	135.8 ± 3.5	130.8 ± 3.7
	End	144.2 ± 4.1 ^BC^	173.8 ± 7.6 ^A$^	135.6 ± 2.7 ^C^	155.7 ± 5.8 ^AB^*	166 ± 6.3 ^A$^	159.4 ± 7 ^AB$^
Tri	Baseline	72.9 ± 3.7	80.1 ± 3.7	68.9 ± 1.1	72.1 ± 3.3	72.3 ± 4.8	77.1 ± 2.6
	End	75.6 ± 3.0 ^A^	69.7 ± 2.9 ^AB^*	46.8 ± 4.0 ^C$^	68.7 ± 4.1 ^AB^	68 ± 4.4 ^AB^	62.2 ± 4.2 ^B#^
Total Cholesterol	Baseline	162.2 ± 5.4	137.8 ± 10.4	167.9 ± 5.4	164.1 ± 12.9	153.3 ± 6	139.0 ± 5.6
	End	147.7 ± 20.5	182.0 ± 21.2 ^#^	158.0 ± 8.4	180.2 ± 15.1	193 ± 13 ^#^	174.6 ± 7.8 ^#^
HDL Cholesterol	Baseline	104.9 ± 3.3	92.9 ± 7.7	104.5 ± 3.4	100.9 ± 5.4	102 ± 2.6	96.4 ± 2.6
	End	85.2 ± 9.0 ^B^*	116.6 ± 12.6 ^A^*	105.2 ± 4.6 ^A^	117.3 ± 8.5 ^A^*	129 ± 8.6 ^A^*	109 ± 5.7 ^AB^
LDL Cholesterol	Baseline	41.3 ± 2.5	28.8 ± 2.7	49.4 ± 3.6	36.0 ± 3.7	36.6 ± 4.6	29.7 ± 2.6
	End	33.5 ± 5.4	51.4 ± 9.2 ^$^	43.3 ± 3.9	42.4 ± 6.4	50 ± 10.8	53.6 ± 6.9 ^$^
VLDL Cholesterol	Baseline	14.7 ± 0.8	15.9 ± 0.8	13.8 ± 0.2	14.1 ± 0.7	13.6 ± 0.6	15.3 ± 0.6
	End	15.0 ± 0.7 ^A^	13.9 ± 0.6 ^AB^*	9.2 ± 0.8 ^C$^	13.8 ± 0.8 ^AB^	14 ± 1 ^AB^	12.3 ± 0.8 ^B#^

Abbreviations: LFD, low-fat diet based on TD.08806 (Envigo); HFD, high-fat diet based on TD.06414 (Envigo); HFD + BAS, HFD + 2% (*w*/*w*) cholestyramine; HFD + AA, HFD + 3.0% (*w*/*w*) acetic acid; HFD + BcL, HFD + 0.8% (*w*/*w*) biocomposite; HFD + BcH, HFD + 5.0% (*w*/*w*) biocomposite; Tri, triglycerides; HDL, high-density lipoprotein; LDL, low-density lipoprotein; VLDL, very-low-density lipoprotein. n = 5–9 per group. Different letters represent significant differences between diets at the endpoint by FDR post hoc analysis following two-way repeated measures ANOVA. *, *p* < 0.05, ^#^, *p* < 0.01, ^$^, *p* < 0.001 vs. baseline by FDR post hoc analysis following two-way repeated measures ANOVA. ^1^ Bile acid sequestrant (cholestyramine). ^2^ Acetic acid. ^3^ Chitosan and sepiolite at a 0.8:1 ratio in 1 N acetic acid, resulting in final concentrations of 2.5% sepiolite and 2% chitosan (*w*/*v*).

**Figure A4:**
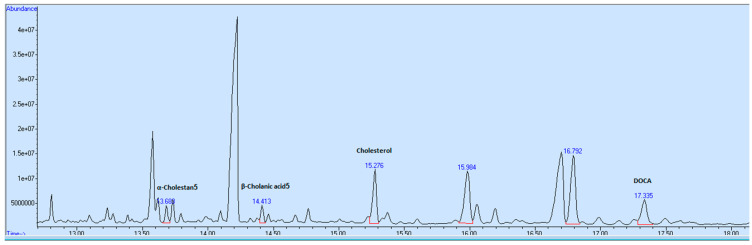
A chromatogram of some of the bile acids.

## Data Availability

The data presented in this study are available upon request from the corresponding author. The data are not publicly available due to that the data are part of an ongoing study.

## Abbreviations

Metabolic syndrome, MetS; nonalcoholic fatty liver disease, NAFLD; bile acid sequestrants, BASs; acetic acid, AA; high-fat diet, HFD; HFD-induced obesity, DIO; biocomposite, Bc; low-fat diet, LFD; Bc low, BcL; Bc high, BcH; fat mass, FM; fat-free mass, FFM; nuclear magnetic resonance, NMR; total energy expenditure, TEE; high-density cholesterol, HDL-C; low-density cholesterol, LDL-C; very-low-density cholesterol, VLDL-C; gas chromatography–mass spectrometry, GCMS; false discovery rate, FDR; cholic acid, CA; lithocholic acid, LCA; deoxycholic acid, DCA; chenodioxycholic acid, CDCA.

## Appendix A

**Table A1:** The effect of diet on key characteristics (mean ± SD).

Parameter	LFD	HFD	HFD + BAS ^1^	HFD + AA ^2^	HFD + BcL ^3^	HFD + BcH ^3^
Change in body weight (g)	1.4 ± 1.8 ^C^	10.9 ± 1.6 ^A^	0.8 ± 1.5 ^C^	5.7 ± 3.2 ^B^	8.9 ± 5.3 ^A^	4.4 ± 2.1 ^B^
Change in fat percentage (% points)	−0.8 ± 4.4 ^C^	28.0 ± 9.2 ^A^	−2.5 ± 6.2 ^C^	17.2 ± 13.7 ^AB^	28.0 ± 10.1 ^A^	12.4 ± 10.2 ^B^
Energy expenditure (Kcal/d/g)	0.6 ± 0.1 ^B^	0.6 ± 0.1 ^B^	0.7 ± 0.1 ^A^	0.6 ± 0.1 ^B^	0.6 ± 0.1 ^B^	0.7 ± 0.1 ^A^
Hepatic lipids (% vs. LFD)	100.0 ± 27.8 ^B^	171.2 ± 43.3 ^A^	119.8 ± 13.6 ^B^	181.2 ± 21.1 ^A^	159.2 ± 24.7 ^A^	105.3 ± 29.9 ^B^

Abbreviations: LFD, low-fat diet based on TD.08806 (Envigo); HFD, high-fat diet based on TD.06414 (Envigo); HFD + BAS, HFD + 2% (*w*/*w*) cholestyramine; HFD + AA, HFD + 3.0% (*w*/*w*) acetic acid; HFD + BcL, HFD + 0.8% (*w*/*w*) biocomposite; HFD + BcH, HFD + 5.0% (*w*/*w*) biocomposite. n = 5–9 per group. Different letters represent significant differences between diets by FDR post hoc analysis following one-way ANOVA. ^1^ Bile acid sequestrant (cholestyramine). ^2^ Acetic acid. ^3^ Chitosan and sepiolite at a 0.8:1 ratio in 1 N acetic acid, resulting in final concentrations of 2.5% sepiolite and 2% chitosan (*w*/*v*).
